# Mindshift in autism: a call to professionals in research, clinical, and educational settings

**DOI:** 10.3389/fpsyt.2023.1251058

**Published:** 2023-08-31

**Authors:** Alana J. McVey, Desiree R. Jones, T. C. Waisman, Dora M. Raymaker, Christina Nicolaidis, Brenna B. Maddox

**Affiliations:** ^1^Department of Psychiatry and Behavioral Sciences, University of Washington, Seattle, WA, United States; ^2^Seattle Children’s Autism Center, Seattle, WA, United States; ^3^Department of Psychology, School of Behavioral and Brain Sciences, University of Texas at Dallas, Richardson, TX, United States; ^4^Autism Training Academy, Vancouver, BC, Canada; ^5^School of Social Work, Regional Research Institute for Human Services, Portland State University, Portland, OR, United States; ^6^Department of Medicine, Oregon Health & Science University, Portland, OR, United States; ^7^Department of Psychiatry, TEACCH Autism Program, University of North Carolina at Chapel Hill, Chapel Hill, NC, United States

**Keywords:** autism, stigma, discrimination, neurodiversity, disability justice

## Abstract

Autistic people often have poor outcomes over the life course, including in health, education, employment, and community inclusion. Many professionals working with Autistic adults in research, clinical, and educational settings devote their careers to trying to improve such outcomes. However, we maintain that real progress cannot happen without a fundamental mindshift. The status quo for professionals is to view autism as an illness. Instead, the neurodiversity movement encourages us to value and embrace autism as an aspect of human diversity and asks us to view Autistic people as a marginalized group that experiences significant disparities. While some professionals may be adopting language and concepts from the neurodiversity movement, we argue that making this mindshift fundamentally changes our practice across research, clinical, and educational settings. In this perspective, we call on professionals to embrace this mindshift to reduce discrimination and stigma, halt the spread of harmful ideologies, and help Autistic adults live fulfilling lives.

## 1. Introduction

Autistic adults are a marginalized group of people that experiences discrimination and stigma (1, 2). Like other marginalized populations, these factors lead to poor outcomes in health, education, employment, quality of life, and community inclusion (3–7). However, researchers, clinicians, and educators—as well as the general public—usually frame autism *itself* as the poor outcome rather than Autistic people as a disparity group (8, 9). Doing so can reinforce ableist views and result in even more discrimination, stigmatization, misrepresentation, dehumanization, abuse, harm, and traumatization (8, 10–12). Viewing autism as an illness can additionally communicate that Autistic people are inferior to allistic [i.e., non-Autistic neurodivergent and neurotypical people; (13)], which may result in internalized ableism in Autistic people (1, 2, 6, 11, 12, 14).

For years, Autistic self-advocates have attempted to de-pathologize autism through the neurodiversity movement (14, 15), which applies the social model of disability to reframe autism as an aspect of human diversity. Neurodiversity is defined as “variation in neurocognitive functioning” [Hughes (16), 3 as cited by Kapp (15)], and neurodivergence includes autism, attention-deficit/hyperactivity disorder (ADHD), and learning disabilities, among others (15). Thus, neurodiversity defines a group of people comprised of different neurotypes. Neurodivergent is defined as “having a mind that functions in ways which diverge significantly from the dominant society standards of ‘normal’” [Walker (17) as cited by Bertilsdotter Rosqvist et al. (18)] and thus refers to an individual. The neurodiversity movement “advocates for the rights of neurodivergent people, applying a framework or approach that values the full spectra of differences and rights such as inclusion and autonomy” [(15), 2]. This model asks us to switch our frame from autism as a deficit or pathology (15, 19) to Autistic adults as a marginalized population that experiences discrimination. This perspective allows for the needs of all Autistic adults (i.e., across all levels of support need and intellectual ability) to be viewed without discrimination or judgment (15) and highlights that all Autistic adults have a right to accommodations, supports, equitable access to society, and a high quality of life. This does not mean that autism is not a disability—Autistic self-advocates commonly identify autism as a disability [e.g., (20)]; this mindshift merely changes the way we view the needs of Autistic people from a medical model (where the individual is flawed and must be fixed) to a social one [where the setup of the environment determines whether a person struggles or succeeds; see Kapp (15)].

Professionals working with Autistic adults in research, clinical, and educational settings have a duty to make this mindshift to reduce discrimination and stigma, halt the spread of harmful ideologies, and acknowledge the trauma Autistic adults experience in academic and medical systems. Doing so fundamentally changes the way we conduct our work across research, clinical, and educational settings.

The purpose of this perspective is to describe the changes in our practice that result from embracing the paradigm of neurodiversity across research, clinical, and educational settings. We happily recognize the growing number of Autistic researchers, clinicians, and educators; and we primarily direct our recommendations toward allistic allies. In forming these recommendations, we bring professional experience in research (clinical and developmental psychology, public health, medicine, mental health services, systems science, implementation science, and community-based participatory research), clinical practice (clinical psychology and internal medicine), post-secondary education, and leadership. We also bring our personal lived experiences as Autistic, otherwise neurodivergent, or neurotypical people, family members, and activists.

## 2. Mindshift in action

### 2.1. Reframing goals

When we hold neurodiversity in mind, we are shifting our mental framework from fixing the Autistic person to helping them achieve a high quality of life. In research settings, this affects the questions we ask (e.g., “how can we remove systemic barriers for Autistic people?” instead of, “how can we change Autistic people to ‘fit’ into existing systems?”), the outcomes we measure (e.g., increased well-being instead of a reduction of autistic traits), and the grant funding sources we pursue (e.g., those that promote neurodiversity framing, include Autistic reviewers, support research conducted by Autistic scholars, and/or provide support for authentic Autistic engagement in the research). Within the clinic, this framework affects our case conceptualization [e.g., trauma-informed and strengths-based; (21, 22)], our treatment targets (e.g., driven by the client’s wishes, focused on promoting well-being as defined by the client), and our approach with clients [e.g., focus on treating co-occurring conditions and increasing function as opposed to treating autism itself; (20)]. Utilizing a collaborative goal setting model, such as shared decision making (23), with Autistic clients and, if applicable, their caregivers, can help us achieve these objectives as clinicians. As educators, the neurodiversity paradigm affects our educational support targets (e.g., encouraging personal interest and inspiration for learning through student-centered engagement and expression) as well as our measures of student progress and program success (e.g., evaluating student growth in knowledge and understanding over time, evaluating student preparation for next-level courses and/or job readiness).

### 2.2. Viewing supports and accommodations as a human right

From a neurodiversity-affirming perspective, we view supports and accommodations as a human right. That is, each Autistic person needs their own unique and tailored supports to achieve their goals. In this way, as researchers, we are likely to frame our research questions around the barriers and facilitators that hinder or support a high quality of life, to examine the effect of supports and accommodations, and to understand how barriers can be reduced or eliminated. As clinicians, we view supports and accommodations as falling under the purview of the Americans with Disabilities Act (24) and work to identify and provide appropriate, tailored, and responsive supports and accommodations for the clients with whom we work (25). This might include providing advance preparations for an office visit (26); considering sensory needs and adjusting our setting appropriately [e.g., dimming lights or providing natural lighting, ensuring access to a quiet space (27)]; changing how we communicate with clients to prioritize their receptive or expressive communication needs; using strategies to help clients tolerate examinations and procedures; supporting clients’ need for consistency or challenges with executive function; and considering the best way to incorporate caregivers while encouraging client-autonomy and shared-decision-making (25, 28). The AASPIRE Healthcare Toolkit^1^ includes tools and resources to help healthcare providers make individualized accommodations and may help improve client-provider communication and reduce barriers to care (28). Within an educational setting, this means facilitating access to appropriate supports and accommodations for students in our classrooms and laboratories as well as bolstering their own self-advocacy (29). Because the process of obtaining accommodations can serve as a barrier to access for many Autistic students (30, 31), it may also be helpful to incorporate principles from Universal Design for Learning [UDL; (32, 33)] to create a more equitable learning environment. Although more research is needed to clarify the efficacy, scope, and implementation of UDL (34, 35), helpful strategies might include providing course materials in multiple formats, including written captions and alt text for videos and images, supplementing in-person handouts with online pdf versions, allowing for alternative participation modalities, providing written feedback at regular points throughout the semester, and including links and information related to accessibility resources within the course syllabi (33, 36). In addition to these strategies, further empirical work evaluating the efficacy of inclusive teaching practices, such as those outlined through validation theory (37), community pedagogy (38), and inclusive pedagogy (39), may help to develop “best practices” for supporting neurodivergent students in the classroom. Across these settings, our willingness to offer support and accommodations ensures Autistic people can more readily access clinical services and education and meaningfully participate in research.

### 2.3. Valuing Autistic people’s lived experience

Marginalizing a group, by definition, de-centers that group’s sources of knowledge; specifically, those with power to legitimize knowledge use it to devalue and dismiss the lived experience of the marginalized group while continuously reinforcing their own power and knowledge (40, 41). To disrupt this mechanism, it is essential to center Autistic people’s lived experience as not just legitimate but as the central or primary source of knowledge about autism.

Taking a neurodiversity frame means centering Autistic people’s lived experiences by listening to them, seeking to understand them, valuing them, and—crucially—actively rejecting conflicting narratives from those who do not have the legitimacy of lived experience no matter how powerful. In research, this means asking Autistic people what they would like to have researched and how and, if need be, pivoting research agendas to those priorities. Some scaffolding to do this in research includes using emancipatory research approaches to assemble teams that meaningfully include Autistic scientists and Autistic community members, ensuring their voices are prioritized as they play an active role throughout the entire research process, and compensating them fairly for their contributions (42–45). The practice of emancipatory participatory research further safeguards that the research is relevant to the Autistic community (9). Other scaffolding to do this work well and do it safely includes training researchers (including on how to work with diverse Autistic adults in trauma-informed and psychologically safe ways), providing adequate supports for Autistic co-researchers and collaborators, and securing sufficient funding (46). Further, valuing Autistic people’s lived experience in research means providing the necessary accommodations to obtain direct report data from them, not from proxy reporters. We provide extensive resources for autism researchers who wish to use participatory approaches and create accessible study materials at www.aaspire.org/inclusion-toolkit.

Within clinical practice, we encourage providers to understand their client’s whole and unique lived experiences, which requires humility, an awareness of intersectionality—that is, the unique experiences of those with multiple marginalized identities (47)—and a responsive style. A Rogerian person-centered approach may be helpful for promoting clinician authenticity, empathy, and positive regard (48). Within educational settings, valuing lived experiences includes training, hiring, and supporting Autistic educators, including Autistic co-facilitators and guest lecturers, involving Autistic people in curriculum development, and including written works by Autistic authors on course syllabi (49–51). Educators may also implement student-centered teaching approaches, creating an accepting environment where students feel comfortable sharing their emotions and experiences (52, 53). By creating space for students to voice their individual needs and concerns, and providing positive feedback, educators can also help to build confidence in Autistic students who have previously faced invalidation (37).

### 2.4. Using neurodiversity affirming language

One aspect of this mindshift is reflected in the language we use to talk about autism. Historically, language pertaining to autism has been largely informed by the medical model, but recent literature points to the need for autism researchers to move away from harmful, ableist language, and instead, center Autistic people’s needs, preferences, and lived experiences (13). Bottema-Beutel et al. (54) as well as Botha et al. (10) provide detailed descriptions on how this can be accomplished. To briefly summarize, Bottema-Beutel et al. (54) ask autism researchers to identify language that may be patronizing, deficit-based, or otherwise ableist and replace it with nonableist terminology (e.g., specifically describing a behavior is an alternative to the term “challenging behavior” or using “co-occurring” instead of “co-morbid”). We believe these recommendations can and must be applied to clinical and educational settings as well by using nonableist and nonstigmatizing terms in spoken and written materials (e.g., therapy handouts or worksheets, course lectures and materials). When conducting autism diagnostic evaluations in clinical settings, this may include describing a client’s challenges rather than their “deficits” (21). To take this even further, a clinician may consider how communication challenges may have more to do with the dynamic interaction between clinician and client, rather than a “deficit” seated within the Autistic person (55). Across research, clinical, and educational contexts, identity-first language (“Autistic person” as opposed to person-first language, “person with autism”) is aligned with the neurodiversity movement, *and* it is important to note that there are individual differences in preferences [e.g., (56–58)]. We recommend using each person’s preferred terminology.

### 2.5. Working within fundamentally ableist systems

As individuals, shifting our mental frame away from a medicalized way of viewing autism toward a social justice model affects our work, but does not in and of itself remove us from the fundamentally ableist systems in which we work. There are opportunities, however, to push back against and innovate these systems. As researchers, we advocate for community-driven research that centers autistic lived experience, reflects community priorities, and authentically includes Autistic people as both co-researchers and as research participants; further, the commitment to centering autistic priorities means doing so every time, including ending lines of research the community repeatedly has noted as harmful or ethically problematic (e.g., studies with potential for eugenic consequences). As clinicians, although we may be tethered to the Diagnostic and Statistical Manual of Mental Disorders [DSM-5-TR; (59)] for diagnostic purposes, we limit the inclusion of discriminatory and stigmatizing language within assessment reports and provide our clients and their families with explanations as to why this language is used (e.g., insurance requirements). We also routinely identify and name ableism and openly discuss it with clients as we talk with them about their health, healthcare, or wellbeing. Even though the educational classification system is focused on deficits, as educators, we can work to ensure that services are delivered in a manner that affirms diversity and makes learning accessible to all students (39, 60). Additionally, we offer emotional support, validation, and advocacy when indicated for Autistic students who are navigating these ableist systems; we also design our own classes to be universally accessible and to promote a culture of access, such that we reduce the burden of self-advocacy for all our students.

We also advocate directly for systems-level change. Within research, this may consist of requiring the inclusion of Autistic people on research teams, in the peer-review process, or on funding boards (43); requiring stringent reporting of conflicts of interest (61); and providing Autistic community members opportunities to voice their concerns without fear of retaliation. For clinicians, we advocate for clinic and/or hospital polices that allow for neurodiversity-affirming practices and documentation. As educators, we support trainings led by Autistic faculty, staff, and students to identify and understand their needs as well as promote autism knowledge and acceptance (62–64).

### 2.6. Leveraging greater systems change

Systems thinker Donella Meadows provides a framework for identifying and understanding leverage (i.e., places where a small change can create a large impact) within systems. The first level of leverage in the framework includes adjustments in numbers, buffers, and materials, such as increasing the number of Autistic scientists, clinicians, and educators, or increasing the capacity of research, clinical, or educational systems to support neurodiversity approaches. The next level of leverage changes the nature of relationships within a system (but not the system’s structure itself), such as modifying how we use language on clinical reports or strengthening the connections between the Autistic community and the research community through participatory research models. These two levels of leverage, as outlined in the previous section, provide ways to push back against existing systems, and we are starting to see evidence of their success (65).

However, as we move into the future, it is both a challenge and an opportunity to consider how we can move beyond existing ableist systems and invoke the next two levels in the framework to remove sources of stigma all together. At the third level of the framework sits leverage that modifies the structure of the system—the flow of information through the system (including who can access it), the rules of the system, and the very way that the system is constructed (66). One place to look to for ideas in implementing interventions at this level of leverage is the Sins Invalid Disability Justice framework (67), which—contrasted with traditional disability rights that advocates inclusion within existing systems—encourages new structures to emerge from within Disability culture itself. Focusing on interdependence, intersectionality, and the inherent strengths, values, and resources of the community, Disability Justice provides a roadmap to creating inherently anti-ableist systems.

At the final level of Meadows’ framework is leverage related to whole-system mindshifts. In order of least to greatest impact, they are, “3. [changing t]he goals of the system; 2. [changing t]he mindset or paradigm out of which the system—its goals, structures, rules, delays, parameters—arises; and 1. the power to transcend paradigms (66).” It is in this spirit that we encourage you to think about the potential for a neurodiversity mindshift. What happens when the goal is not normalization or even inclusion but celebration of Autistic bodyminds? What happens when our worldview inherently values neurodivergence? What happens when we have dismantled ableist systems of oppression to the point where Autistic people are no longer discriminated against at all?

## 3. Conclusion

In this perspective, we have shared practical considerations for the ways in which adopting the framework of neurodiversity shifts our work across research, clinical, and educational settings. Understanding that Autistic adults are a marginalized group of people that experiences discrimination and harmful outcomes drives us to shift our frame of mind from one based on a deficit model to one focused on centering the voices of Autistic people and providing appropriate supports and accommodations to help them thrive. Certainly, these recommendations are not a panacea, and there are many barriers to Autistic adults not addressed here. Nonetheless, we hope that you will consider how adopting the neurodiversity paradigm may help you to make immediate, tangible, and helpful changes to the way you conduct your research, interact with and support your clients, and engage and support students in their educational attainment.

## Data availability statement

The original contributions presented in the study are included in the article/supplementary material, further inquiries can be directed to the corresponding author.

## Author contributions

AM, DJ, DR, CN, and BM contributed to the conception of the manuscript. AM wrote the first draft of the manuscript. AM, DJ, TW, DR, and CN wrote sections of the manuscript. All authors contributed to the article and approved the submitted version.

## Conflict of interest

The authors declare that the research was conducted in the absence of any commercial or financial relationships that could be construed as a potential conflict of interest.

## Publisher’s note

All claims expressed in this article are solely those of the authors and do not necessarily represent those of their affiliated organizations, or those of the publisher, the editors and the reviewers. Any product that may be evaluated in this article, or claim that may be made by its manufacturer, is not guaranteed or endorsed by the publisher.

## Footnotes

^1^www.autismandhealth.org
